# Genomic and Metabolomic Profile Associated to Clustering of Cardio-Metabolic Risk Factors

**DOI:** 10.1371/journal.pone.0160656

**Published:** 2016-09-02

**Authors:** Vannina G. Marrachelli, Pilar Rentero, María L. Mansego, Jose Manuel Morales, Inma Galan, Mercedes Pardo-Tendero, Fernando Martinez, Juan Carlos Martin-Escudero, Laisa Briongos, Felipe Javier Chaves, Josep Redon, Daniel Monleon

**Affiliations:** 1 Metabolomic and Molecular Image Lab, Health Research Institute, INCLIVA, Valencia, Spain; 2 Genotyping and Genetic Diagnosis Unit, Health Research Institute, INCLIVA, Valencia, Spain; 3 Department of Nutrition, Food Science and Physiology, University of Navarra, Pamplona, Spain; 4 INCLIVA Research Institute, University of Valencia, Valencia, Spain; 5 Internal Medicine, Hospital Rio Hortega, Valladolid, Spain; 6 CIBERDem, Health Institute Carlos III, Madrid, Spain; 7 CIBERObn, Health Institute Carlos III, Madrid, Spain; University of Nebraska Medical Center, UNITED STATES

## Abstract

**Background:**

To identify metabolomic and genomic markers associated with the presence of clustering of cardiometabolic risk factors (CMRFs) from a general population.

**Methods and Findings:**

One thousand five hundred and two subjects, Caucasian, > 18 years, representative of the general population, were included. Blood pressure measurement, anthropometric parameters and metabolic markers were measured. Subjects were grouped according the number of CMRFs (Group 1: <2; Group 2: 2; Group 3: 3 or more CMRFs). Using SNPlex, 1251 SNPs potentially associated to clustering of three or more CMRFs were analyzed. Serum metabolomic profile was assessed by ^1^H NMR spectra using a Brucker Advance DRX 600 spectrometer. From the total population, 1217 (mean age 54±19, 50.6% men) with high genotyping call rate were analysed. A differential metabolomic profile, which included products from mitochondrial metabolism, extra mitochondrial metabolism, branched amino acids and fatty acid signals were observed among the three groups. The comparison of metabolomic patterns between subjects of Groups 1 to 3 for each of the genotypes associated to those subjects with three or more CMRFs revealed two SNPs, the rs174577_AA of FADS2 gene and the rs3803_TT of GATA2 transcription factor gene, with minimal or no statistically significant differences. Subjects with and without three or more CMRFs who shared the same genotype and metabolomic profile differed in the pattern of CMRFS cluster. Subjects of *Group 3* and the AA genotype of the rs174577 had a lower prevalence of hypertension compared to the CC and CT genotype. In contrast, subjects of *Group 3* and the TT genotype of the rs3803 polymorphism had a lower prevalence of T2DM, although they were predominantly males and had higher values of plasma creatinine.

**Conclusions:**

The results of the present study add information to the metabolomics profile and to the potential impact of genetic factors on the variants of clustering of cardiometabolic risk factors.

## Introduction

Clustering of cardiometabolic risk factors (CMRFs) such as abdominal obesity, high fasting glucose, elevated blood pressure, elevation of triglycerides and reduced HDL levels, is a frequent condition, and when three or more of these conditions are present, this has been named the Metabolic Syndrome (MS). It is a highly prevalent condition, with an estimated quarter of all adults having three or more CMRFs, and its presence increases the risk to develop type 2 diabetes as well as the risk for cardiovascular morbidity and mortality [1–3]. Although the MS concept has been challenged, as clustering does not provide more risk than the sum of the individual risk factors, assessment of the clustering of CMRFs is still a useful tool in clinical practice to recognize patients at risk [4].

Factors related to the development of the clustering of CMRFs factors are not well understood, but it is thought to be the interplay of genetic and environmental factors. The relative impact of each of them on the development of the MS components was studied by Poulsen et al [5] in a total of 303 elderly twin pairs. The concordance rates for glucose intolerance, overall obesity and low HDL-cholesterol were significantly higher among monozygotic than dizygotic twins indicating a genetic influence on the development of these phenotypes. In contrast, the heritability estimates for waist-to-hip ratio, fasting insulin, triglycerides and blood pressure were low, indicating a major environmental influence. As a complex trait, several genes can participate in the development, forming a pleiotropic intertwined genetic network [6]. Genome-wide association studies (GWAS) have identified several genes associated with the clustering of CMRFs, including genes involved in lipid metabolism (CETP, APOA1/C3/A4/A5 cluster, LPL, LIPC and ABCB11), glucose sensing (GCKR), insulin signaling (IRS1), beta‐cell function (TCF7L2), and appetite control (FTO). However, these variants explain only a small fraction of the observed heritability [7], and the relevance of each genetic variant, or others that may act in concordance, is not well understood (Song et al, 2006).

Recently, studies have applied the metabolomic approach to identify a discriminatory metabolite profile in a large number of diseases [8–11]. Metabolomics, which attempts to capture global changes and overall physiological status in biochemical networks and pathways, can be useful in order to elucidate sites of perturbations and has shown great promise as a means to identify biomarkers [12] even at early stages of disease. Likewise, it may be useful for understanding metabolic imbalances and for detecting previously unsuspected links to pathological conditions [11,13]. Abnormalities in metabolomic profile have been described in subjects with clustering of CMRFs [14–16]; however, no studies have considered the information provided for the interplay between genomics and metabolomics.

In order to better understand the role of genetics in this complex trait disease and to look for potential early identification of people with higher genetic risk to develop it, the objective of the present study was to identify links between genetic markers and metabolomic profile in subjects with a clustering of CMRFs.

## Materials and Methods

### Study Population

The study was performed in subjects from a population-based study in which the selection criteria and methodology have been previously described [17]. Briefly, the sample included individuals older than 18 years in the absence of serious concomitant disease or psychiatric disorder, which could interfere with the study. All the subjects included were white, living in an area with a low immigration rate. To be representative of the general population, investigators calculated the sample size by using local public resources and finally 1502 subjects were included. From all the patients studied, 1213 with a high genotyping call rate were analysed. The study was approved by the local Ethics Committee of Hospital Clinico Universitario de Valencia, INCLVIA Research Institute, Valencia, and informed consent forms were signed by all of the subjects prior to participation in this study. Participants gave their informed consent to use their blood samples for genetic studies.

The population included in the present study is the same that has been used in a previous study of our group in which the risk of microalbuminuria was presented *(Marrachelli VG*, *Monleon D*, *Rentero P*, *Mansego ML*, *Morales JM*, *Galan I*, *Segura R*, *Martinez F*, *Martin-Escudero JC*, *Briongos L*, *Marin P*, *Lliso G*, *Chaves FJ*, *Redon J*. *Genomic and metabolomic profile associated to microalbuminuria*. *PLoS One*. *2014 Jun 11;9(2)*:*e98227*. *doi*: *10*.*1371/journal*.*pone*.*0098227)*. The data presented are different to those in the referenced manuscript since the issue analysed is unrelated, the former assessed the risk of microalbuminuria and the present the risk to develop metabolic syndrome unrelated to the microalbuminuria issue.

### Assessment of Metabolic Syndrome Components and Other Cardiovascular Risk Factors

The study included the assessment of anthropometric measurements, blood pressure, glycaemia, lipid profile and smoking status as well as personal and familial information about cardiovascular risk factors and disease. Cardiometabolic risk factors were identified, according to the ATPIII criteria used for MS [18], and MS was defined by the presence of three or more of the following components: 1) high waist circumference (men ≥ 102cm; women ≥88 cm); 2) high triglycerides (≥150mg/dL); 3) low HDL cholesterol (men ≥ 40mg/dL; women ≥50mg/dL); 4) high blood pressure (systolic blood pressure ≥130 mmHg and/or diastolic blood pressure ≥ 85 mmHg or being on antihypertensive medications) and 5) high fasting glucose (≥ 110 mg/dL or being on drug treatment for elevated glucose). The subjects were divided into three groups: *Group 1* comprised of 617 subjects with less than two risk criteria of the ATPII guideline; *Group 2* comprised of 295 subjects with 2 risk factors and *Group 3* comprised of 283 subjects with 3 or more of the criteria, which is considered to be MS. Weight was assessed with precise scales while the individuals were without shoes and wearing light clothing. Height was determined in a similar way. Body mass index (BMI) was calculated using the following formula "weight (kg)/height^2^ (m)". Glucose and lipid profile was measured in blood samples obtained with a mean of 3 hours fasting (range 0–17). Basic serum biochemistry and lipid profile (total cholesterol, HDL cholesterol and triglycerides) were measured in Hitachi 917 autoanalyzer (Boehringer, Germany). Blood pressure was measured using a mercury sphygmomanometer following the recommendations of the British Hypertension Society. Systolic BP (SBP) and diastolic BP (DBP) were the average of 3 readings measured at 5-minute intervals.

### Single-Nucleotide-Polymorphism Selection and Genotyping

One thousand two hundred and fifty one single nucleotide polymorphisms (SNP) potentially associated to metabolic risk components were selected based on a bibliography search and those frequency described in the dbSNP database for a Caucasian population. These include genes involved in lipid metabolism, oxidative stress, mitochondrial respiratory chain, renin-angiotensin system and other biological processes. Genotyping was carried out by using SNPlex (Applied Biosystems, Foster City, California, USA).

### NMR Spectroscopy

Eighty-two microliters of D2O were added to 418 μl of blood serum and placed in a 5-mm NMR tube. ^1^H NMR spectra were recorded using a Bruker Avance DRX 600 spectrometer (Bruker GmbH, Rheinstetten, Germany). Samples were measured at 37°C. Nominal temperature of the sample was kept at 37°C. A single-pulse pre-saturation experiment was acquired in all samples. The spectra were referenced using the doublet of Alanine at 1.478 ppm. The chemical shift region, including resonances between 0.50 and 4.70 parts per million of spectrometer frequency (ppm), was investigated. The spectra were binned into 0.01 ppm buckets and normalized to total aliphatic spectral area to eliminate differences in metabolite total concentration. Signals belonging to selected metabolites were quantified using semi-automated in-house MATLAB 6.5 (The MathWorks Inc., Natick, Massachusetts) integration and peak-fitting routines. Reproducibility of NMR spectroscopy was tested by superposition of normalized spectra of blood serum. Chenomx NMR Suite 4.5 software and two-dimensional NMR methods including homonuclear correlation spectroscopy (TOCSY) and heteronuclear single quantum correlation spectroscopy (HSQC) were used to identify and subsequently confirm the assessment of metabolites.

Chemometric statistical analyses were performed using in-house MATLAB scripts and the PLS Toolbox (Eigenvector Research, Inc.). Principal Components Analysis (PCA) was performed after data was pretreated by mean centering and Pareto scaling. A PLS-DA model discriminating between group 1 and group 3 was constructed. The multivariated chemometric models were cross-validated with 10-fold Venetian blind cross-validation; in each run, 10% of the data was left out of the training and used to test the model. The whole cross validation process was run 10 times. The results of cross validation were evaluated by the Q2 (R2CV) and RMSCV parameters. Q2 is the average correlation coefficient between the dependent variable and the PLS-DA predictions and provides a measure of prediction accuracy during the cross-validation process (higher values mean better prediction). Root Mean Square Error of Cross-Validation (RMSCV) was calculated as an adequate measurement of over fitting. Permutation test was also performed for testing for over-fit regression models (Random t-test) as well as for providing a probability that the given model is significantly different from one built under the same conditions but on random data. Score plots were used to visualize the separation of the groups, while the variable importance in the projection (VIP) value of each variable in the model was calculated to indicate its contribution to the classification. A higher VIP value represented a stronger contribution to discrimination among groups. VIP values >1.0 were used to determine which spectral variables significantly contributed to the separation of the samples on the score plots. Fold change was calculated by dividing the mean metabolite concentration in *Group 3* minus *Group 1* divided by *Group 3*.

### Statistical Analysis

All values are expressed as mean ± SD. The χ^2^ goodness-of-fit test was used to compare the distribution of the study population. Genotypes and allele frequencies were calculated for every SNP. The Hardy-Weinberg equilibrium was sought by a χ^2^-distribution with one degree of freedom. Those SNPs that were not in Hardy-Weinberg equilibrium and did not have more than 90% of genotyping were excluded from the subsequent analysis. The Hardy-Weinberg equilibrium was calculated using PLINK (http://pngu.mgh.harvard.edu/~purcell/plink/). The association of MS with each polymorphism was performed using PLINK by logistic regression models. The mean differences of the two groups’ p-values were tested against a conservative Bonferroni p-threshold for α = 0.05 experiment-wise, which corresponded to p = 1.85e-04 for 27 tests. MS associations were tested by linear regression models.

The metabolomic profiles of patients of *Group 1* and *Group 3* were compared. The association between metabolic profile and genetic variants was sought by using the loading plots of the metabolic discriminating PLS-DA model for each selected SNP genotype. For more accurate characterization of each metabolite association with the SNPs, we calculated the difference of the relative metabolic levels’ average between *Group 1* and *Group 3* patterns for each polymorphism normalized to the same differences at global levels, irrespective of genotype. Differences in the 28 metabolite values for each SNP in patients from *Group 1* and *Group 3* of each genotype were calculated. Finally, the metabolic profile and the most relevant metabolites of each genotype and allele were compared between patients from *Group 1* and *Group 3*. The data were co-variated with respect to age, sex and smoking status. Bonferroni correction was applied in all the analysis. Statistical analyses were performed using the IBM SPSS Statistics 19 software.

## Results

### General Characteristics of the Study Population

The general characteristics of the 1213 subjects grouped by the number of CMRFs are in Table 1. Thirty-two percent of subjects had two CMRFs, and 23% had three or more. Subjects in Group 3 were older compared to the other two groups. As expected, there was a progressive increment in the prevalence of diabetes, hypertension and abdominal obesity from *Group 1* to *Group 3*. Likewise, BMI, fasting glucose, systolic and diastolic BP and triglycerides were also higher and HDL lower in *Group 3* compared to the other groups.

**Table 1:** General characteristics of the study population grouped by CMRFs.

		Group 1	Group 2	Group 3
Number of samples		596	290	281
Sex, n (%)	Male	276 (46.3%)	168 (28.2%) ^a^	152 (25.5%) ^a^
	Female	320 (56.0%)	122(21.4%) ^a^	129 (22.6%) ^a^
Age (years)		47 ± 18	57 ± 19^a^^,^^b^	67 ± 15^a^
BMI (kg/m^2^)		24 ± 3	27 ± 4^a^^,^^b^	30 ± 4^a^
SBP (mmHg)		122 ± 17	134 ± 20^a^^,^^b^	146 ± 21^a^
DBP (mmHg)		75 ± 9	81 ± 10^a^^,^^b^	86 ± 10^a^
Glycemia (mg/dl)		86 ± 11	93 ± 17^a^^,^^b^	109 ± 30^a^
Creatinine (mg/dl)		0.8 ± 0.2	0.9 ± 0,4	0,9 ± 0,6^a^
Total Cholesterol (mg/dl)		194 ± 34	205 ± 39^a^	211 ± 39^a^
LDL (mg/dl)		111 ± 33	118 ± 35^a^	116 ± 36
HDL (mg/dl)		58 ± 12	47 ± 12^a^^,^^b^	43 ± 11^a^
LogTG (mg/dl)		2.1 ± 0.2	2.3 ± 0.2^a^^,^^b^	2.4 ± 0.2^a^
Diabetes mellitus 2		11 (1.8%)	20 (6.9%) ^a^^,^^b^	67 (23.8%) ^a^
Hypertension		134 (22.5%)	135 (46.6%) ^a^^,^^b^	224 (79.7%) ^a^
Abdominal obesity		178 (29.9%)	157 (54.1%) ^a^^,^^b^	190 (67.6%) ^a^
Obesity		42 (7.0%)	85 (29.3%) ^a^^,^^b^	156(55.5%) ^a^
HTN treatment		62 (10.4%)	59 (20.3%) ^a^^,^^b^	109 (38.8%) ^a^
DM treatment		6 (1.0%)	10 (3.4%) ^a^^,^^b^	36 (12.8%) ^a^
HCT-TG treatment		25 (4.2%)	18 (6.2%) ^b^	36 (12.8%) ^a^

^a^ statistically different from Group 1;

^b^ Statistically different from Group 2.

### CMRFs and SNPs Polymorphism

From the total 1251 SNPs tested, six polymorphisms on 4 genes were significantly associated with the presence of at least three CMRFs: *FADS2* (rs174577 and rs174589), *GSR* (rs 2978663), *GATA 2* (rs3803), *TFAP2B* (rs2272903). The main characteristics of the SNPs and the degree of association are shown in Table 2. No associations remained significant after Bonferroni correction (Bonferroni-corrected p = 0.000039).

**Table 2:** SNPs associated to CMRFs in the general population.

Chr	SNP	Model	Gene	Location	Gene description	p-value
11	rs174589		FADS2	intron variant	Fatty acid desaturase 2	0.00027
11	rs174577		FADS2	intron variant	Fatty acid desaturase 2	0.00078
8	rs2978663		GSR	intron variant	glutathione-disulfide reductase	0.00037
3	rs3803		GATA2	utr variant 3 prime	GATA binding protein 2—TF	0.00079
6	rs2272903		TFAP2B	utr variant 5 prime	transcription factor AP-2 beta	0.00086

### CMRFs and Metabolomic Profile

**Fig 1:**
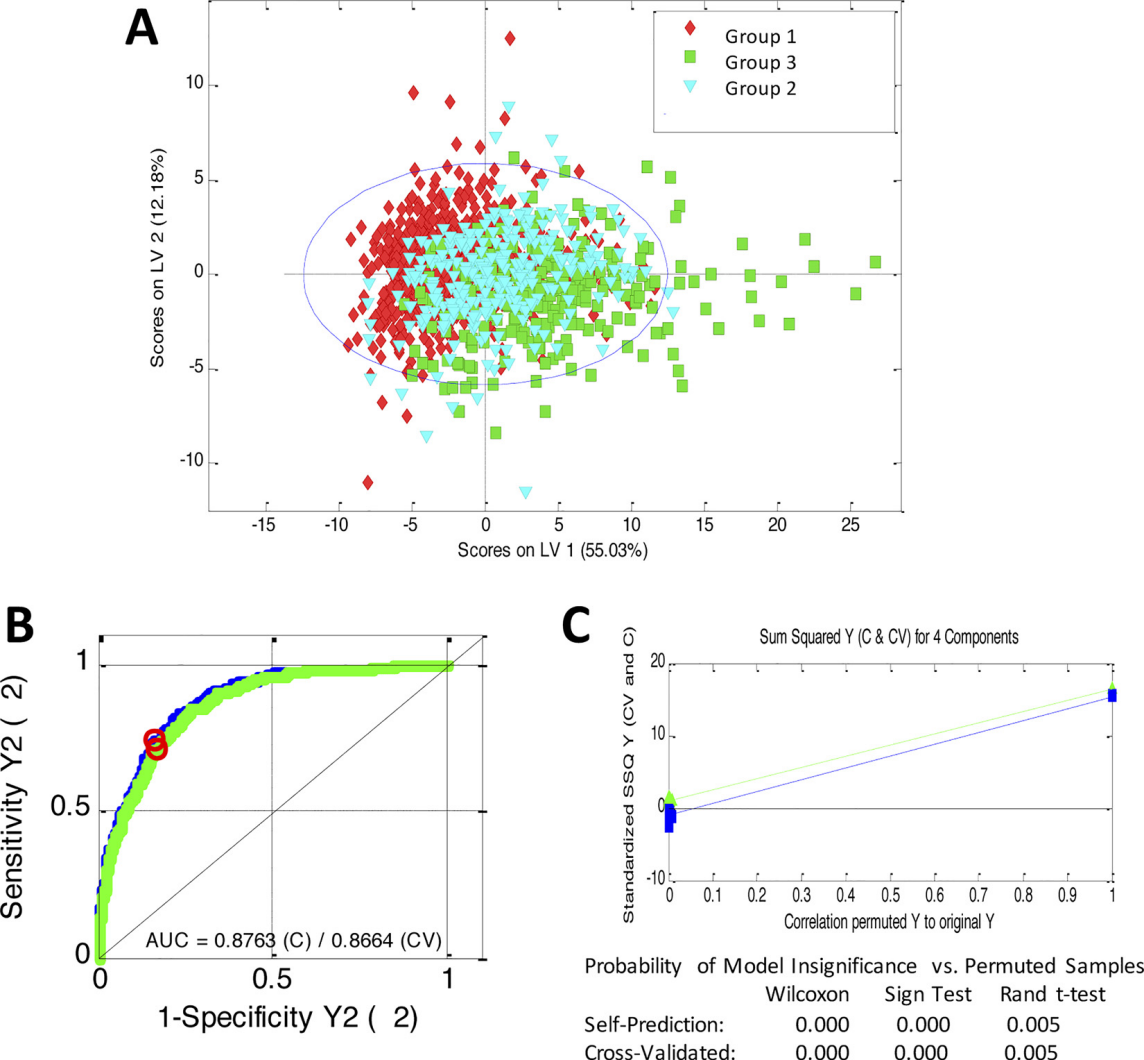
PLS-DA model scores plot (A) for discrimination between subjects from Group 1 (red circles) and Group 3 (close circles) based on the NMR spectra of blood serum of the entire cohort. The permutation tests were carried out with 100 random permutations in PLS-DA models. Cross-validated receiver operating characteristic (ROC) curve (B) showing the prediction capacity of the model, with an area under the curve of 0.876. Permutation analysis of PLS-DA model (C) derived from subjects group 1 versus subjects from group 3. Statistical validation of the PLS-DA model by permutation analysis using 50 different model permutations. The Probability of Model Insignificance vs. Permuted Samples is shown below.

**Fig 2:**
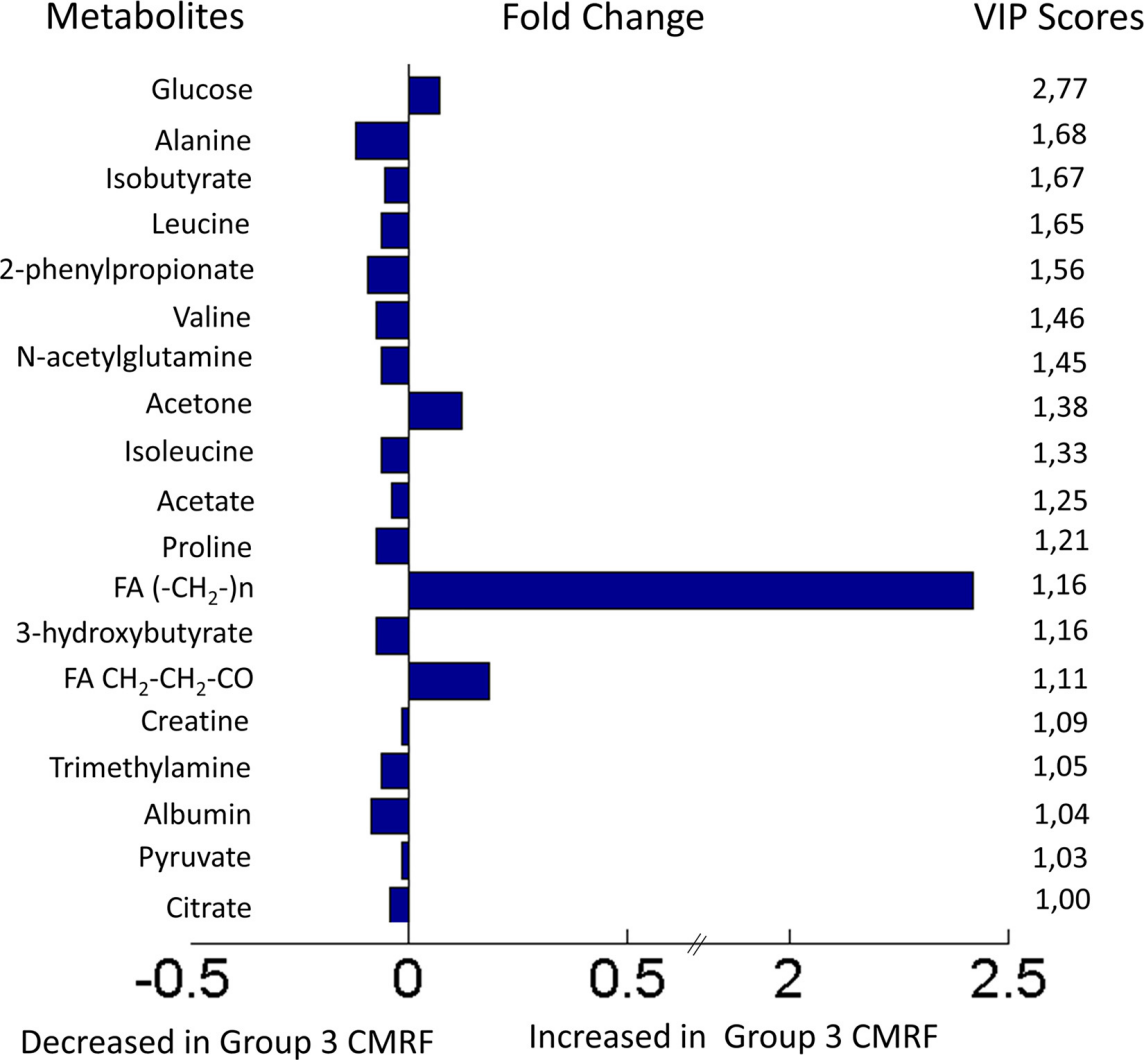
Key metabolite differences subjects from group 1 and from group 3 in the whole population.

Principal component analysis (PCA) was initially performed with the normalized peak areas obtained from all the samples to evaluate the quality of sample analysis and to view the holistic distribution, clustering, and outlier of samples. The PCA scores plot shows that most of the samples in the study are tightly clustered in a small area, indicating that the current protocol is reliable and thereby the variance derived from metabolomic analysis can be ignored at the following data analysis. Then, partial least squares discriminant analysis (PLS-DA) was applied. The PLS-DA model showed significantly improved goodness of fit, adequate model predictability, and fairly good capability to explain the metabolic variation between subjects from *Group 1* and *Group 3* (Fig 1A). Samples from the different groups were well separated along the first PLS components, which indicates that NMR- based metabolic profile could reveal characteristic alterations in plasma from subjects from *Group 3* compared to the other two groups. Permutation testing and cross validation, two established methods of internal validation, were used to confirm model validity. Permutation tests involve the random assignment of class labels to cases and controls. Permutation testing using 50 random permutations demonstrates that the goodness of fit (RMSCV = 0.84) and predictive ability (Q2 = 0.30 and an accuracy of 88%) of the original model discriminating groups (Fig 1C) was higher than those of the permuted models. Using cross-validated Y-predicted values, model sensitivity and specificity were summarized using ROC curves for the model distinguishing Group 1 (AUROC = 0.8763) from Group 3 subjects (Fig 1B). Results were indicative of quite a strong predictive power. After spectral integration, differences were observed among patients in Group 1 and Group 3 (Fig 2). As shown in Table 3, the differential endogenous compounds detected included mitochondrial metabolism (citrate), extra mitochondrial metabolism (glucose, pyruvate, lactate, creatinine, creatine, creatine phosphate) and several amino acids and their derivative signals (such as proline, glutamine, N-acetylglutamine, alanine, tyrosine, tryptophan). Among these, branched amino acids (valine, isoleucine, leucine) exhibited a relatively high statistical significance. We also detected fatty acid signals, (FA-CH2-CH2CO, FA(-CH2-)n, FA-CH2-CH3), as well as signals from cholesterol, phosphoethanolamine, choline, isobutyrate, 3-hydroxybutyrate, trimethylamine, methanol, acetone, acetate, 2-phenylpropionate and albumin.

**Table 3:** Metabolite relative levels in serum from subjects group 1 versus subjects from group 3.

Metabolites	ppm	Group 1	Group3	P-value
Cholesterol	0.60–0.75	3.53 ± 0.49	3.24 ± 0.46	<0.00001
Lipid (-CH_3_)	0.80–0.90	8.06 ± 0.39	8.11 ± 0.38	1.23E-01
Leucine	0.95–0.97	1.02 ± 0.05	0.95 ± 0.06	<0.00001
Isoleucine	0.99–1.02	0.99 ± 0.04	0.95 ± 0.05	<0.00001
Valine	1.02–1.05	1.10 ± 0.05	1.03 ± 0.07	<0.00001
Isobutyrate	1.05–1.07	0.71 ± 0.04	0.66 ± 0.05	<0.00001
Lipids (-CH_2_-)_n_	1.18–1.31	14.05 ± 1.98	16.47 ± 2.76	<0.00001
Lactate	1.32–1.35	2.02 ± 0.41	2.34 ± 0.47	<0.00001
2-phenylpropionate	1.40–1.44	1.35 ± 0.07	1.26 ± 0.10	<0.00001
Alanine	1.46–1.50	1.66 ± 0.08	1.54 ± 0.12	<0.00001
Lipids (CH_2_-CH_2_-CO)	1.55–1.60	1.85 ± 0.16	2.03 ± 0.22	<0.00001
Acetate	1.91–1.92	0.56 ± 0.03	0.52 ± 0.05	<0.00001
N-acetylglutamine	1.92–1.95	0.92 ± 0.05	0.86 ± 0.06	<0.00001
Lipids (-CH_2_CH_3_)	1.95–2.03	3.77 ± 0.17	3.91 ± 0.22	<0.00001
Acetone	2.21–2.23	0.64 ± 0.09	0.76 ± 0.14	<0.00001
Pyruvate	2.35–2.36	0.18 ± 0.02	0.17 ± 0.02	<0.00001
Citrate	2.50–2.54	0.46 ± 0.04	0.41 ± 0.05	<0.00001
Lipids (= CH-CH_2_-CH =)	2.71–2.80	1.25 ± 0.09	1.26 ± 0.10	9.48E-01
Trimethylamine	2.9–2.95	0.66 ± 0.06	0.60 ± 0.07	<0.00001
Proline	3.30–3.35	0.34 ± 0.05	0.32 ± 0.05	<0.00001
Methanol	3.35–3.36	0.08 ± 0.01	0.07 ± 0.02	5.70E-03
Creatine	3.91–3.92	0.17 ± 0.01	0.16 ± 0.02	<0.00001
Tyrosine	3.92–3.94	0.27 ± 0.02	0.25 ± 0.03	<0.00001
Creatine-P	3.94–3.95	0.17 ± 0.01	0.15 ± 0.02	<0.00001
O-Phosphoethanolamine	3.95–3.99	0.61 ± 0.05	0.57 ± 0.06	<0.00001
Creatinine	4.03–4.05	0.31 ± 0.02	0.30 ± 0.02	2.84E-05
Tryptophan + choline	4.05–4.07	0.33 ± 0.02	0.35 ± 0.03	<0.00001

### Metabolomic Profile, Selected Genotypes and CMRFs

**Fig 3:**
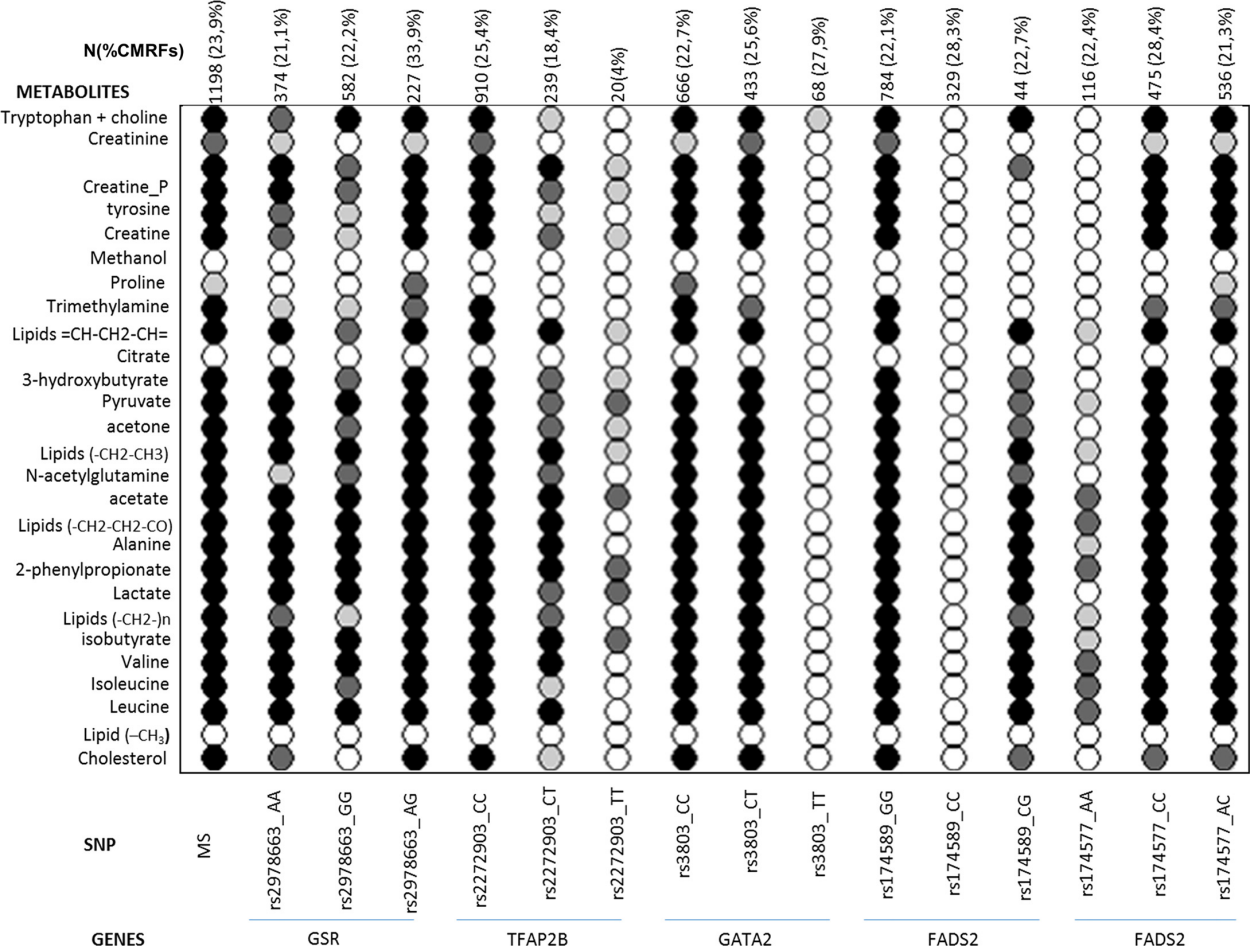
Patterns of statistical significance, calculated as p-values, for the comparison of metabolic profiles from subjects group 1 versus subjects from group 3 in the whole population (first column) and in individuals with different SNPs (rest of the columns). (white) p> 0.01; (white-gray) p< 0.01; (gray) p< 0.001; (black) p< 0.00001.

**Fig 4:**
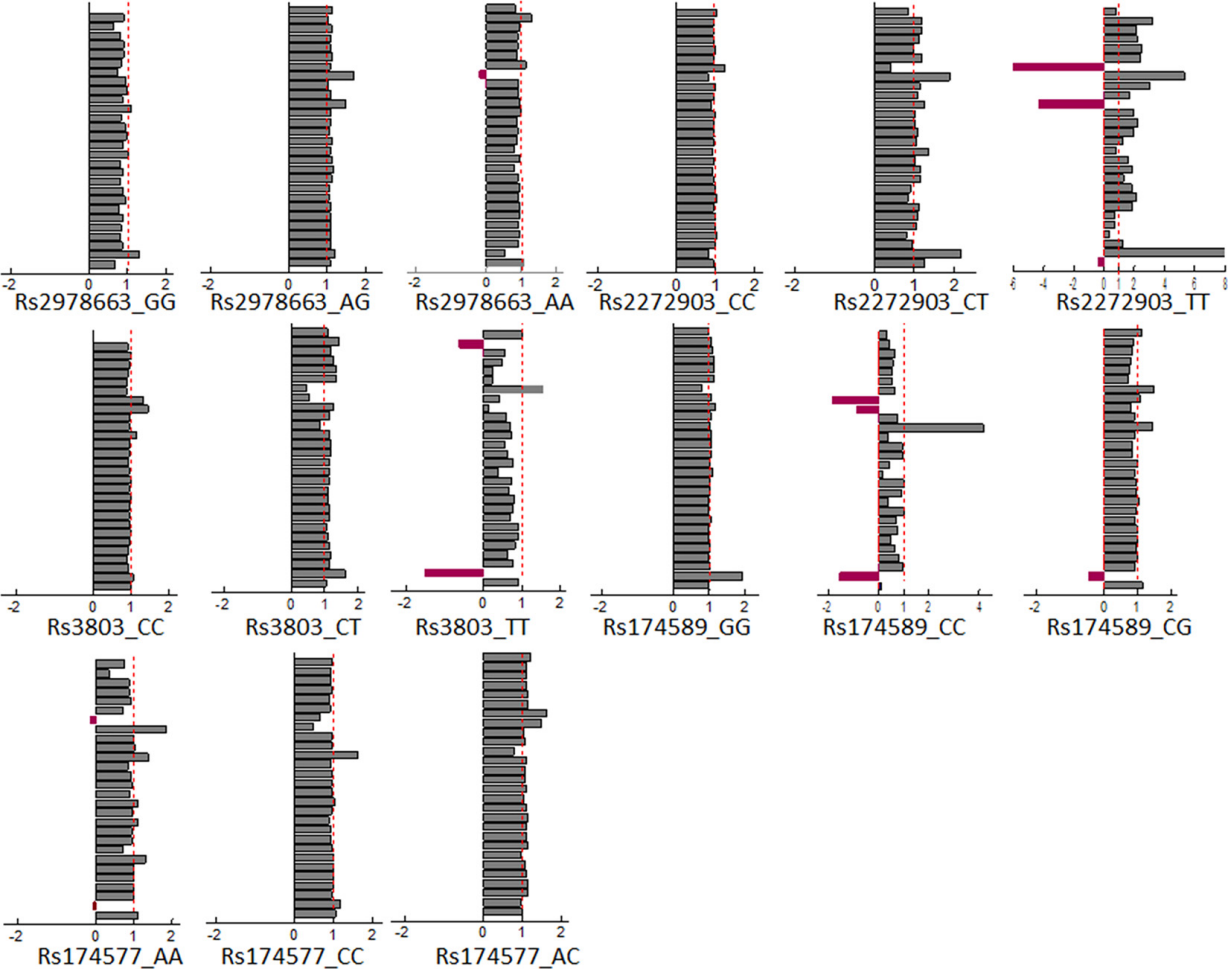
Bar chart showing metabolic differences between Group 3 and Groups 1 normalized with respect to changes in group 3 (see Table 1). The bars represent the difference in the average metabolic levels between Group 3 and group 1 for each SNP divided by the same difference calculated for the entire cohort. SNPs with bars closer to 1 (dotted line) show CMRFs associated metabolic changes similar to those of the global population (irrespective of genotype). On the other hand, SNPs with bars closer to 0 exhibit minimal or no metabolic changes associated to CMRFs. Bars with negative values indicate a CMRF associated metabolic change opposite to that detected in global population. Metabolites from top to bottom are: tryptophan + choline; creatinine; phosphoethanolamine; creatine phosphate; tyrosine; creatine; methanol; proline; trimethylamine; lipids (= CH-CH2-CH2 =); citrate; 3-hydroxybutyrate; pyruvate; acetone; lipids (-CH2-CH3); N-acetylglutamine; acetate; lipids (-CH2-CH2_CO); alanine; 2-phenylpropionate; lactate; lipids (-CH2-)n; isobutyrate; valine; isoleucine; leucine; lipids (-CH3) and cholesterol.

The metabolomic profiles of the genotypes of the 4 SNPs associated to three or more CMRFs were obtained. In each of these genotypes, we compared the metabolomic profile between subjects from Group 1 and those from Group 3 with three or more CMRFs and those with less. The values for each metabolite were tested for statistical significance between Group 1 and Group 3 both as a whole and at the individual SNP level (Fig 3). The comparison of the statistical significance patterns revealed four SNPs (rs2272903_TT of the TFAP2B gene; rs3803_TT of the GATA 2 gene; rs174589_CC and rs 174577_AA of the FADS2 gene) with minimal or no statistically significant differences between three or more CMRFs and a metabolic profile with very few differences between those with three or more, and less status (Fig 4). Genotypes rs2272903_TT and rs174589_CC were considered to have too low a sample count and they were excluded for further analysis. Then, we looked for the characteristics of the subjects from *Group 1* and *Group 3* who shared the same genotype and the same metabolomic profile. Subjects from *Group 3* and of the AA genotype of the rs 174577 had a lower prevalence of hypertension (15 subjects, 57.7%) compared to the CC (95 subjects, 83.3%) and CT (109 subjects, 80.7%) genotype (Table A in S1 File). Subjects from *Group 3* and of the TT genotype of the rs3803 polymorphism, 19 subjects, had a lower prevalence of DM2 (5.3% *vs* 24.2%; p = 0.009) compared to the CC genotype (151 subjects, 25.8%) although they were predominantly male and had higher values of plasma creatinine (1.37 ± 0.2 mmol/L *vs* 0.91 ± 0.24 mmol/L) (Table B in S1 File).

## Discussion

Metabolomic profiling of clustering of CMRFs has recently opened up new expectations for better detection, characterization and stratification of the patient. In the present study we analyzed a general Spanish population and identified an MS metabolomic profile associated to changes in amino acid metabolism, glucose homeostasis, lipid β-oxidation, tricarboxylic acid (TCA) cycle, urea cycle and microbiota-host co-metabolism. The study identified two genes in which subjects with a given genotype did not show differences in the metabolic profile between subjects from Group 1 and Group 3, and that differed in the pattern of CMRFs cluster. While the subjects with at least three CMRFs and the TT genotype of the rs3803 had a very low prevalence of diabetes and obesity, those with the AA genotype of the rs174577 had less prevalence of hypertension and higher prevalence of low HDL.

The study was performed in subjects, representative of the general population from an area with a low rate of external admission. Almost a quarter of the adults in this study had a diagnosis of MS, which is in agreement with other population-based studies in Spain [3]. Our cohort exhibited a BMI that seems slightly lower than that reported for the cohorts in other studies with Spanish populations [19]. The average BMI of the subjects in *Group 3* was 30±4, indicating a general population with overweight or moderate obesity, but not very severely obese as described elsewhere [20]. Subjects in *Group 3* were weakly associated to genotypes of SNPs located in the chromosomes 3, 6, 8 and 11. These SNPs were located mainly in genes related to atherosclerosis and obesity such as FADS2 (rs174577 and rs174589), GATA 2 (rs3803) and TFAP2B (rs2272903). However, the degree of association in some of them was not high enough to be considered a positive association per se, and therefore data from metabolomics was used to improve the capacity of analysis. Data from the metabolomic study provides further insight into the potential relationship between genotypes and the clustering of CMRFs.

Metabolomics provides a powerful approach to identifying biomarkers caused by both genetic and non-genetic factors, by analyzing global changes in an individual’s metabolic profile even at early stages of disease. In this study, an RMN-based serum metabolomics approach, coupled with multivariate statistical methods, provides a powerful approach which allows for discrimination between patients with three or more, and less CMRFs, and the identification of potential biomarkers. The good match between the results in training and cross-validation datasets provides further support to the model. PLS-DA revealed an evident and statistically significant separation between *Group 1* and *Group 3* (leaving group 2 between them), thus suggesting that metabolomics may unravel metabolic differences before they become clinically or biochemically evident.

The differential metabolomic profile shows that branched amino acids (BCAA) are reduced in MS. BCAA can act as signalling molecules in many processes. The combined effect of lipids and BCAA seems pivotal in a complex network of interactions involving muscle, adipose, liver and brain metabolisms [21]. Although some studies report increased BCAA levels in diabetes and insulin resistance, the role of these metabolites in cardiometabolic diseases is still controversial. In rodents, diet-induced insulin-resistance and obesity are associated with a decrease in BCAA serum levels [22]. Previous clinical studies also showed that hypertensive patients suffer from depleted proteins stores [23,24]. Diet, exercise and basal metabolism strongly affect BCAA levels [25] and preclude an explanation of the findings.

The metabolic changes observed in patients with at least three CMRFs are numerous and of complex interpretation. Changes in lipids, glucose, pyruvate, lactate, alanine and glutamine suggest shifts in energy metabolism. Choline, which is also altered, has a predominant role in cell membrane integrity, methyl metabolism and lipid-cholesterol transport [26]. Cardiovascular risk factor profile has been associated with high choline in plasma suggesting a disruption of choline oxidation to betaine as part of the mitochondrial dysfunction [27].

Acetate is a final product of lipid metabolism and can be converted into acetyl-Coenzyme A (acetyl-CoA) by acetyl-CoA synthetase. It can also be related to acetone by the spontaneous decarboxylation of acetoacetate, which may explain up to 11% of gluconeogenesis in fasting obese subjects [28]. However, in addition to the complex network of interactions among the different metabolites due to the host metabolism, it is necessary to take into account the co-metabolism with the gut microbiota. Acetate, propionate and n-butyrate, altered in *Group 3* and related to the metabolites mentioned above, are the most important short chain fatty acids (SCFAs) produced during fermentation by gut bacteria [26]. These findings, combined with the observation of decreased TMA and methanol in *Group 3* subjects, suggest a potential role for microbiota co-metabolism in the development of CMRFs in our population.

The combined analysis of -omics data represents a highly challenging task in the analysis of clinical samples. Although systems biology may provide useful models for prediction of system response to particular perturbations, the application to the analysis of multicellular organisms is not exempt from difficulties. The analysis of multi -omic data in patient cohorts by molecular stratification represents a practical approach for better characterization of the disease. We performed a genotype stratified metabolomic analysis of CMRF status. This allowed us to detect genotypes with atypical CMRF metabolomic profiles. For example, we detected four polymorphisms in which the metabolomic impact of the clustering of CMFRs is different to that of the global cohort. These polymorphisms (FADS2 rs174577 and rs174589, GATA2 rs3803 and TFAP2B rs2272903) affect genes mostly associated to lipid metabolism.

Δ-6-fatty acid desaturase (FADS2) is the key enzyme in the biosynthesis of polyunsaturated fatty acids (PUFAs). We report that individuals with the rs174577_AA in the same gene have larger Group 3 changes in proline and no changes in methanol and some fatty acids. The strong association between FADS genotype and fatty acid levels in our data is in line with previous studies and suggests a role in lipid homeostasis for this gene [29–31]. FADS gene cluster polymorphisms are associated to HOMA-IR in healthy men [32]. The lipid changes observed in our metabolomic data may be related to those observed in FADS2-deficient mice, which in turn are obesity-resistant [31]. However, the effects of FADS2 on plasma lipid profiles are very variable since differences in the dietary intake of polyunsaturated fatty acids may be responsible for this variability and increase the complexity of the analysis [33]. The different profiles for these two polymorphisms have a reflection in their phenotype. Hypertension is less prevalent in *Group 3* patients with the rs174577_AA polymorphism with a higher component of metabolic abnormalities.

GATA2 transcription factor (rs3803) plays a key role in adipogenesis. Our stratified metabolomic analysis reveals that having at least three CMRFs affects the fatty acids profile differently in individuals with this genotype. Constitutive GATA-2 expression suppresses adipocyte differentiation and traps cells at the preadipocyte stage [34]. This effect is mediated through the direct suppression of peroxisome proliferator-activated receptor gamma PPARγ 2 [34] and the interaction of GATA factors with C/EBP [35]. In our population, individuals from *Group 3* and this genotype had a lower prevalence of DM2 and obesity.

## Conclusions

The results of the present study add information to the metabolomics profile and to the potential impact of genetic factors on the variants of clustering of cardiometabolic risk factors. The global metabolomic profile of subjects with three or more CMRFs was also present in individuals without them but with specific genotypes. These individuals have different clinical characteristics, which suggest that those genotypes may represent distinct phenotypes of the clustering of MS and may help in the better stratification of these patients. Moreover, the metabolomic profile is similar in those with or without the minimal clustering of risk factors to fulfill the criteria of MS, indicating the potential risk to develop in the future and/or the necessity for additional interventions to prevent development and reduce cardiovascular morbidity and mortality. The potential risk of the different clustering can result in more selective interventions tailored according to the main risk of each genotype.

## Supporting Information

S1 File Table A: General characteristics of subjects with Group 1 and Group 3 for the genotype of the rs174577. Table B: General characteristics of subjects with Group 1 and Group 3 for the genotype of the rs3803.
(DOCX)
