# Supplementary material for: Genomic and Metabolomic Profile Associated to Clustering of Cardio-Metabolic Risk Factors
Source: PLoS One. 2016 Sep 2;11(9):e0160656. doi: 10.1371/journal.pone.0160656 (PMC5010244; doi:10.1371/journal.pone.0160656)
Supplement: S1 File — Table A: General characteristics of subjects with Group 1 and Group 3 for the genotype of the rs174577. Table B: General characteristics of subjects with Group 1 and Group 3 for the genotype of the rs3803. (DOCX) [file pone.0160656.s001.docx]

|  | | | **rs174577** | | | | | |
| --- | --- | --- | --- | --- | --- | --- | --- | --- |
|  | | | **AA** | | **AC** | | **CC** | |
|  | | | **Group1** | **Group3** | **Group1** | **Group3** | **Group1** | **Group3** |
| **N of samples** | | | 90 | 26 | 340 | 135 | 422 | 114 |
| **Sex (M/F)** | **Male** | | 45(50)  45(50) | 15(57,7)  11(42,3) | 162(47,6)  178(52,4) | 76(56,3)  59(43,7) ^a^ | 222(52,6)  200(47,4) | 57(50)  57(50) |
|  | **Female** | |  |  |  |  |  |  |
| **Age** | | | 51 ± 19 | 67 ± 18^a^ | 51 ± 18 | 66 ± 15^a^ | 49 ± 19 | 67 ± 15^a^ |
| SBP | | | 126 ± 19 | 143 ± 21^a^ | 126 ± 20 | 144 ± 21^a^ | 125 ± 19 | 150 ± 22^a, b^ |
| **DBP** | | | 76 ± 9 | 83 ± 11^a^ | 77 ± 10 | 85 ± 11^a^ | 77 ± 10 | 87 ± 11^a^ |
| **BMI** | | | 25 ± 3 | 29 ± 5^a^ | 24 ± 6 | 30 ± 5^a^ | 25 ± 3 | 30 ± 4^a^ |
| **glycemia** | | | 87 ± 14 | 103 ± 21^a^ | 89 ± 14 | 114 ± 36^a^ | 87 ± 15 | 105 ± 23^a, b^ |
| **Total cholesterol** | | | 202 ± 42 | 212 ± 53 | 197 ± 35 | 209 ±39^a^ | 198 ± 39 | 211 ± 36^a^ |
| **LDL** | | | 114 ± 36 | 117 ± 44 | 114 ± 32 | 113 ± 37 | 113 ± 35 | 119 ± 34 |
| **HDL** | | | 54 ± 15 | 39 ± 8^a^ | 54 ± 13 | 42 ± 12^a^ | 55 ± 13 | 44 ± 10^a^ |
| **Log TG** | | | 2,17 ± 0,21 | 2,39 ± 0,24^a^ | 2,12 ± 0,19 | 2,39 ± 0,20^a^ | 2,12 ± 0,20 | 2,36 ± 0,18^a^ |
| **HTN** | | | 26 (28,9) | 15 (57,7) ^a,b^ | 109 (32,1) | 109 (80,7) ^a , c^ | 125 (29,6) | 95 (83,3) ^a, c^ |
| **DM2** | | | 4 (4,4) | 4 (15,4) | 13 (3,8) | 37(27,4) ^a^ | 14 (3,3) | 25 (21,9) |
| **HTG** | | | 43 (49,4) | 17 (73,9) ^a^ | 135 (41,4) | 103 (87,3) ^a^ | 169 (42,6) | 82 (82,8) ^a^ |
| **HCT_LDL** | | | 11 (12,2) | 8 (30,8) ^a^ | 45 (12,9) | 27 (20) ^a^ | 79 (18,8) | 26 (22,8) |
| **Abdominal obesity** | | | 38 (42,2) | 18 (69,2) ^a^ | 122 (35,9) | 93 (68,9) ^a^ | 162 (38,4) | 77 (65,8) ^a^ |
| **Obesity** | | | 12 (13,8) | 11 (42,3) ^a^ | 50 (15,8) | 74 (54,8) ^a^ | 58 (14,3) | 66 (58,4) ^a^ |
| **Number cardiometabolic risk factors** | | **0-1** | 56 | - | 219 | - | 284 | - |
|  |  | **2** | 34 | - | 112 | - | 126 | - |
|  |  | **≥3** | - | 26 | - | 132 | - | 112 |
| **tto_HTN** | | | 13(14,4) | 10 (38,5) ^a^ | 48 (14,1) | 50 (37,0) ^a^ | 58 (13,7) | 48 (42,1) ^a^ |
| **tto_DM** | | | 2 (2,2) | 2 (7,7) | 7 (2,1) | 22 (16,3) ^a^ | 7 (1,7) | 12 (10,5) ^a^ |
| **tto_HCT-TG** | | | 3 (3,) | 3 (11,5) | 14 (4,1) | 17 (12,6) ^a^ | 24(5,7) | 15 (13,2) ^a^ |

**S1 Table. General characteristics of subjects with Group 1 and Group 3 for the genotype of the rs174577**

S1 Table legend

a statistically different from Group1in the same genotype

b statistically different from Group3 in general population

C stastistically different from Group3 with AA genotype

S2 Table. General characteristics of subjects with Group 1 and Group 3 for the genotype of the rs3803

|  | | | **rs3803** | | | | | |
| --- | --- | --- | --- | --- | --- | --- | --- | --- |
|  | | | **CC** | | **CT** | | **TT** | |
|  | | | **Group1** | **Group3** | **Group1** | **Group3** | **Group1** | **Group3** |
| **N of samples** | | | 515 | 151 | 322 | 111 | 49 | 19 |
| **Sex (M/F)** | **Male** | | 260(50,5) | 79(52,3) | 155(48,1) | 59(53,2) | 29(59,2) | 14(73,7) |
|  | **female** | | 255(49,5) | 72(47,7) | 167(51,9) | 52(46,8) | 20(40,8) | 5(26,3) ^a, b^ |
| **Age** | | | 51 ± 19 | 67 ± 15^a^ | 49 ± 19 | 68 ± 14^a^ | 45 ± 17 | 62 ± 14^a^ |
| SBP | | | 127 ± 20 | 146 ± 21^a^ | 125 ± 18 | 146 ± 21^a^ | 122 ± 14 | 147 ± 23^a^ |
| **DBP** | | | 78 ± 10 | 85 ± 11^a^ | 76 ± 9 | 86 ± 11^a^ | 76 ± 7 | 87 ± 7^a^ |
| **BMI** | | | 26 ± 4 | 30 ± 4^a^ | 24 ± 7 | 29± 4^a^ | 24 ± 7 | 29 ± 3^a^ |
| **glycemia** | | | 88 ± 14 | 110 ± 28^a^ | 87 ± 13 | 110 ± 32^a^ | 89 ± 13 | 98 ± 24 |
| **Total cholesterol** | | | 198 ± 37 | 209 ± 39^a^ | 198 ± 39 | 212 ± 39^a^ | 197 ± 41 | 206 ± 36 |
| **LDL** | | | 113 ± 33 | 115 ± 36 | 114 ± 34 | 115 ± 38 | 112 ± 38 | 122 ± 34 |
| **HDL** | | | 54 ± 13 | 43 ± 11^a^ | 55 ± 14 | 43 ± 11^a^ | 54 ± 12 | 40 ± 8^a^ |
| **Log TG** | | | 2,14 ± 0,20 | 2,38 ± 0,18^a^ | 2,11 ± 0,20 | 2,39 ± 0,21^a^ | 2,13 ± 0,20 | 2,33 ± 0,13^a^ |
| **creatinine** | | | 0,83 ± 0,20 | 0,91 ± 0,24 | 0,86 ± 0,38 | 0,89 ± 0,25 | 0,87 ± 0,17 | 1,37 ± 0,2^b^ |
| **HTN** | | | 178 (34,6) | 124 (82,1) ^a^ | 84 (26,1) | 86 (77,5) ^a^ | 7 (14,3) | 14 (73,7) ^a^ |
| **DM2** | | | 20 (3,9) | 39 (25,8) ^a^ | 10 (3,1) | 27 (24,3) ^a^ | 1 (2) | 1 (5,3) ^b, c^ |
| **HTG** | | | 217 (42,1) | 112 (74,2) ^a^ | 124 (39,7) | 79 (81,4) ^a^ | 19 (39,6) | 14 (73,7) ^a^ |
| **HCT_LDL** | | | 80 (15,5) | 38 (25,2) ^a^ | 49 (15,3) | 23 (19,8) | 9 (18,4) | 2 (10,5) |
| **Abdominal obesity** | | | 202 (39,2) | 101 (66,9) ^a^ | 114 (35,4) | 76 (68,5) ^a^ | 19 (38,8) | 13 (68,5) ^a^ |
| **Obesity** | | | 74 (14,4) | 84 (55,6) ^a^ | 46 (14,8) | 64 (57,7) ^a^ | 7 (15,6) | 8 (42,1) ^a^ |
| **Number cardiometabolic risk factors** | | **0-1** | 344 | - | 218 | - | 34 | - |
|  |  | **2** | 171 | - | 104 | - | 15 | - |
|  |  | **≥3** | - | 151 | - | 111 | - | 19 |
| **tto_HTN** | | | 85 (16,5) | 61 (40,4) ^a^ | 33 (10,62 | 44 (39,6) ^a^ | 3 (6,1) | 4 (21,1) |
| **tto_DM** | | | 12 (2,3) | 16 (10,6) ^a^ | 4 (1,2) | 19 (17,1) ^a^ | 0 (0) | 1 (5,3) |
| **tto_HCT-TG** | | | 33 (6,4) | 22 (14,6) ^a^ | 9 (2,8) | 14 (12,6) ^a^ | 1 (2) | 0 (0) |

S2 Table legend

^a^ statistically different from Group 1in the same haplotype

^b^ statistically different from Group3 in general population

^C^ statistically different from Group3 with CC-genotype
